# Supercritical Antisolvent Precipitation of Corticosteroids/β-Cyclodextrin Inclusion Complexes

**DOI:** 10.3390/polym16010029

**Published:** 2023-12-20

**Authors:** Stefania Mottola, Iolanda De Marco

**Affiliations:** 1 Department of Industrial Engineering, University of Salerno, Via Giovanni Paolo II 132, 84084 Fisciano, SA, Italy; smottola@unisa.it; 2 Research Centre for Biomaterials BIONAM, University of Salerno, Via Giovanni Paolo II 132, 84084 Fisciano, SA, Italy

**Keywords:** corticosteroid, inclusion complex, β-cyclodextrin, SAS micronization, controlled release, supercritical carbon dioxide, supercritical antisolvent

## Abstract

In this study, corticosteroid–β-cyclodextrin (β-CD) inclusion complexes were prepared by using supercritical antisolvent (SAS) precipitation to enhance the dissolution rate of dexamethasone (DEX) and prednisolone (PRED), which are poorly water soluble drugs. The processing of the active principles in the absence of a carrier led to their almost complete extraction (the small amount of obtained material precipitates in the form of crystals). The coprecipitation of the ingredients in the presence of β-CD was investigated at different concentrations, pressures, and molar ratios. For both the corticosteroids, the optimized operating conditions were 40 °C, 120 bar, an equimolar ratio, and a concentration in DMSO of 20 mg/mL; these conditions led to the attainment of microparticles with mean diameters equal to 0.197 ± 0.180 μm and 0.131 ± 0.070 μm in the case of DEX and PRED, respectively. Job’s method confirmed the formation of inclusion complexes with a 1/1 mol/mol ratio. Compared to the pure ingredients, the obtained powders have an improved release rate, which is about three times faster in both cases. The release curves obtained under the best operating conditions were fitted using different models. The best fitting was obtained using the Weibull model, whose parameters are compatible with a combined release mechanism involving Fickian diffusion and controlled release.

## 1. Introduction

Corticosteroids are a class of synthetic or natural hormones and have been a cornerstone of modern medicine for decades [1]. They have found applications in different medical fields ranging from inflammation and immunosuppression to metabolic regulation and asthma management [2]. Corticosteroids have revolutionized the way to approach and manage a multitude of diseases [1]. This introduction explores corticosteroids’ fundamental properties and applications and clarifies their limitations that require micronization or precipitation with a carrier [2].

Corticosteroids are therapeutic agents that reverse the inflammation present in several chronic or infectious inflammatory diseases [2].

Distinct types of corticosteroids have similar pharmacological effects, with differences in the dose administered and the increased systemic effect. In general, corticosteroids present effects in the treatment of many diseases, such as obstructive pulmonary diseases [3], autoimmune diseases [2], inflammatory skin diseases [4], and infections generating a marked reduction in the number and activation of cell infiltrations. In the literature, a wide range of papers report the effects of corticosteroids and their impact on modern medicine.

Among the wide range of corticosteroids available, of particular interest for the effects demonstrated in the treatment of different symptoms are dexamethasone (DEX) [5,6,7] and prednisolone (PRED) [8,9,10]. DEX and PRED are crystalline powders with a whitish appearance, insoluble in water but readily soluble in acetone and alcohol. Moreover, these active principles are light sensitive, necessitating protection from direct exposure.

For these reasons, they are commonly given in the form of micronized powder paired with a hydrophilic carrier that enhances dissolution in a water-based environment while concurrently shielding them from light radiation, and β-cyclodextrin (β-CD) emerges as a carrier well-matched for both objectives.

The distinctive properties of cyclodextrins mean they are extensively utilized as pharmaceutical excipients [11,12]. Cyclodextrins consist of cyclic oligosaccharides comprising various α-D-glucopyranose units, forming molecules shaped like truncated cones with a central hollow space. Their external surface is hydrophilic, while the inner cavity is lipophilic, allowing accommodation of appropriately sized guest molecules. The significance of cyclodextrins, notably β-cyclodextrin, in the pharmaceutical realm lies in their capacity to create inclusion and non-inclusion complexes with a wide array of drugs [13]. This enables advantageous alterations in the physicochemical characteristics of the guest molecule, leading to enhanced solubility, increased bioavailability, regulated dissolution rate, improved stability, and upgraded sensory qualities [11].

Therefore, cyclodextrins are versatile carriers that enable specific designs for drug delivery systems capable of optimizing the desired physicochemical properties and pharmacokinetic parameters.

Diverse micronization methods, such as spray drying, jet milling, ball milling, and emulsion drying [14,15,16,17], have been established in the field. However, these processes pose significant challenges in predicting and regulating the morphology, mean size, particle size distribution (PSD), solvent removal during micronization, and the requirement for high process temperatures.

The limitations of conventional techniques can be overcome by processing the active principles through innovative processes based on the use of supercritical carbon dioxide (scCO_2_). Processes using scCO_2_ are applied in many research areas [18,19,20,21,22]. The utilization of supercritical fluids offers distinct benefits owing to their unique characteristics. Their gas-like viscosity enables deep penetration into various solid substrates, while their liquid-like solvent properties facilitate the solubilization of numerous organic compounds. Moreover, the solvent capabilities can be conveniently fine-tuned by modulating density through simple adjustments in pressure or temperature. Yet, the primary advantage lies in the potential to yield completely solvent-free products. Achieving this is feasible through a straightforward expansion process, eliminating the necessity for additional post-treatment procedures to remove the gas [23].

Among the scCO_2_-based techniques, supercritical antisolvent (SAS) is one of the most commonly employed to produce small particles with a narrow PSDa and without residual solvent in the final product [24,25,26]. The process is based on the antisolvent action of CO_2_ in the supercritical state towards a solute of interest, which is initially dissolved in an organic solvent. The latter must be perfectly miscible with the supercritical CO_2_ under the process conditions. The insolubility of the solute in the formed solvent–antisolvent mixture determines its precipitation in particulate form [27].

Employing the SAS technique in drug/cyclodextrin systems typically results in the formation of micrometric or nanometric particles characterized by uniform size distributions and a spherical shape. These particles exhibit notably accelerated and consistent drug dissolution rates compared to the unprocessed drugs when subjected to in vitro release tests. For example, Franco and De Marco [24] report using the SAS process to produce anti-inflammatory drugs/β-cyclodextrin inclusion complexes. Yan et al. [26] used the supercritical antisolvent technique to obtain baicalein/hydroxypropyl–β-cyclodextrin inclusion complexes to enhance drug solubility and its antioxidant and antibacterial activities.

The intricate process of forming drug/cyclodextrin inclusion complexes within the SAS method remains unexplored in the literature, leaving room for ongoing discussions and further investigation. These complexes might potentially form either within the liquid solution before interacting with the supercritical antisolvent or during the subsequent precipitation phase [11].

The crucial aspect that must be noted is that the complexation of drugs (when it occurs) happens simultaneously with particle precipitation. Moreover, both occurrences bring similar beneficial effects on the resulting formulations by improving the drug’s aqueous solubility and dissolution kinetics [11]. In the literature, there are no documents in which the SAS process has been used to obtain corticosteroid–β-cyclodextrin inclusion complexes.

Therefore, the SAS technique was used in this work to obtain inclusion complexes using DEX and PRED as corticosteroids. For both active ingredients, the effect of the total concentration of solutes in the liquid phase and the active ingredient/cyclodextrin molar ratio was studied to verify the influence of these parameters on the morphology of the particles and the actual formation of inclusion complexes.

## 2. Materials and Methods

### 2.1. Materials

β-cyclodextrin (β-CD, purity 97%) was supplied by Sigma-Aldrich; dexamethasone (DEX) (purity ≥ 98%) and prednisolone (PRED) were provided by Sigma Aldrich (St. Louis, MO, USA) and Farmabios (Gropello Cairoli, Italy), respectively. Dimethyl sulfoxide (DMSO, purity 99.8%) was supplied by Carlo Erba (Cornaredo, Italy). CO_2_ (purity 99%) was provided by Morlando Group s.r.l. (Naples, Italy). Distilled water was produced by using a laboratory-scale distillatory.

### 2.2. Supercritical Antisolvent (SAS) Process

**Figure 1:**
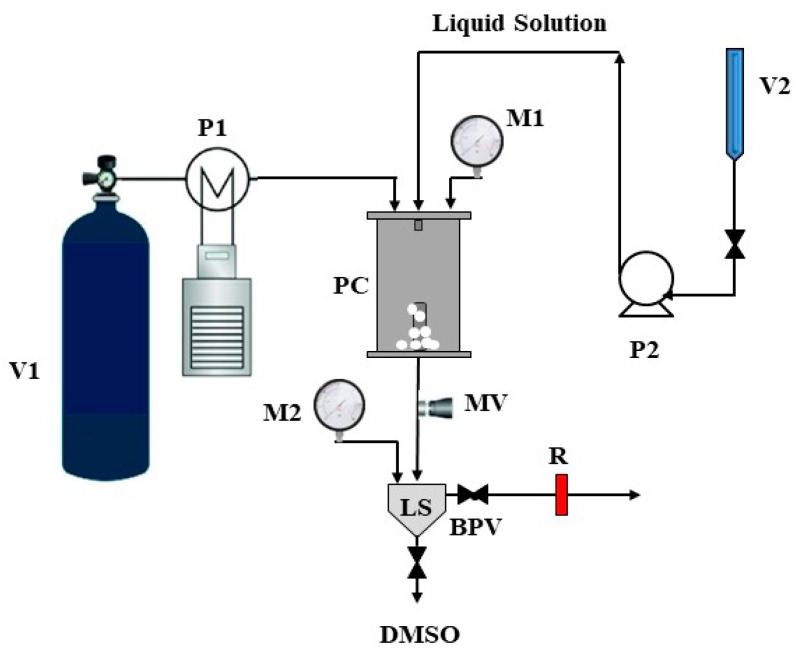
Sketch of the SAS plant. V1: carbon dioxide supply; V2: liquid solution burette; P1: carbon dioxide pump; P2: liquid pump; PC: precipitation chamber; M1 and M2: manometers; MV: micrometric valve; LS: liquid separator; BPB: back-pressure valve; R: rotameter; DMSO: outcoming dimethyl sulfoxide.

The SAS setup, which sketch is reported in Figure 1, consists of a cylindrical vessel with an internal volume of 500 cm^3^, which serves as the principal component of the procedure. Two high-pressure pumps were employed to introduce the carbon dioxide (antisolvent) and the liquid solution (DMSO + solute) into the precipitation chamber (PC) to achieve the necessary pressure. CO_2_ underwent pre-cooling in a refrigerating bath before introduction into the PC, while the liquid solution was injected through a stainless steel nozzle into the PC. A proportional integral derivative (PID) controller and heating bands were used to fix and maintain the PC operating temperature. Pressure regulation was accomplished through a micrometric valve, and a test gauge manometer facilitated pressure measurement.

At the bottom of the PC, a porous filter with 0.1 μm diameter pores allowed the passage of the CO_2_–solvent mixture, enabling the collection of the precipitated powders. The flow rate of CO_2_ was measured using a rotameter. At the onset of each SAS experiment, CO_2_ was pumped into the PC until the desired pressure and temperature were reached. Once the quasi-steady-state composition of the solvent and antisolvent was established, the liquid solution was introduced through the nozzle to start solute micronization. After the solution injection, CO_2_ continued to flow for a calculated duration to ensure the complete removal of solvent residues. Subsequently, the CO_2_ pump was turned off, and the vessel was depressurized gradually to atmospheric pressure.

In the experiments performed during this work, the liquid flow rate was set at 1 mL min^−1^ and the CO_2_ flow rate was 30 g min^−1^. Micronization conditions, including pressure, temperature, and concentration, were chosen in each experiment to understand the effect of these variables on the results. Before each experiment, the solution was prepared by dissolving the solute in 30 mL DMSO, serving as the solvent, to achieve the desired concentration and the maximum sample yield. After each SAS experiment, the powder was collected in the precipitator, and DMSO was extracted using scCO_2_.

### 2.3. Characterization Techniques

#### 2.3.1. FESEM Characterization and PSD Evaluation

The structure of the particles was examined through a field emission scanning electron microscope (FESEM, mod. LEO 1525, Carl Zeiss SMT AG, Oberkochen, Germany). After each experiment, the powder sample collected at the vessel’s base was dispersed on a carbon tab and coated with a thin layer of gold–palladium (layer thickness 250 Å). This preparatory step is crucial to render the samples conductive, enabling observation through microscopy. The acquired images were then processed using image analysis software (Sigma Scan Pro version 5.0.0, Aspire Software International, Ashburn, VA, USA). Approximately 1000 particles were measured to determine average diameters, facilitating the assessment of granulometric distributions of the powders using Microcal Origin Pro 2021 software version 9.8 (OriginLab Corporation, Northampton, MA, USA). The cumulative distributions were obtained by modeling experimental data through log-normal particle size distributions (PSDs).

#### 2.3.2. Fourier Transform Infrared Characterization

Fourier transform infrared (FT-IR) spectra were evaluated using the IRTracer-100 (SHIMADZU Europe, Duisburg, Germany) at a resolution of 0.5 cm^−1^. Next, 100 mg KBr powder was added to 1 mg sample so the disks became transparent to infrared. The mixed powder was compacted into disks with the assistance of a hydraulic press. The analysis was then performed in a scan wavenumber range of 4000 to 500 cm^−1^. Noise decrease was guaranteed by averaging 16 different scanning signals.

#### 2.3.3. X-ray Diffraction

X-ray diffraction analysis (XRD) was performed using an X-ray powder diffractometer (Bruker, Billerica, MA, USA), with the following conditions: Ni-filtered Cu Kα radiation, λ = 1.54 Å, and 2θ angle ranging from 10° to 50°, with a scan rate of 0.5 s/step and a step size of 0.08°.

#### 2.3.4. Dissolution Tests

The UV/vis spectrophotometer (model Cary 60, Varian, Palo Alto, CA, USA) was used to assess the dissolution kinetics of both non-processed and SAS-processed samples. Data for both PRED and DEX were collected at a wavelength of 245 nm. Dissolution tests were conducted in phosphate-buffered saline solution (PBS) at pH 7.4 to mimic the physiological environment. Powders containing a corticosteroid amount equivalent to 5 mg for PRED and 4 mg for DEX were suspended in 3 mL PBS at pH 7.4 inside a dialysis sack. This sack was placed in 300 mL PBS at pH 7.4, stirred continuously at 150 rpm, and maintained at 37 °C.

The release test procedure involved initial sampling at 1 min intervals for the first 100 min, followed by one sample every 5 min for the subsequent 1400 min, and finally, one reading every 30 min until a plateau was reached. At this point, it was assumed that all the drugs had migrated to the outer phase. To ensure result reliability, analyses were performed in triplicate.

Encapsulation yield was determined through UV–vis analysis, measuring absorbance after the release. The absorbance values were then converted into PRED or DEX concentrations using a calibration curve. Loading efficiency was calculated as the ratio between the mean value of effective drug content and the theoretical one.

#### 2.3.5. Dissolution Tests

The drug release data were fitted using Microcal Origin Pro 2021 software (OriginLab Corporation, Northampton, MA, USA) to different kinetic models, such as Korsmeyer–Peppas, Peppas–Sahlin, Higuchi, and Weibull, to understand which represented the correct release mechanism.

#### 2.3.6. Job’s Method

The stoichiometry of the corticosteroid/β-CD inclusion complexes was evaluated using the continuous variation method, also called Job’s plot, named after the author who first used this approach [28]. Two stock solutions with equimolecular concentrations of active pharmaceutical ingredient (API) and β-CD in distilled water were prepared. Subsequently, different volumes of said solutions were mixed to have a constant total concentration (8 × 10^−5^ mmol/mL) and mole fractions of X = [API]/([API] + [β-CD]) varying in the range of 0–1. The samples were sonicated for 15 min and then stirred for three days at constant temperature (25 °C) to reach equilibrium. The solutions were diluted and analyzed with UV/vis spectroscopy at a wavelength of 245 nm. Job plots were then created by mapping ΔA × X against X, where ΔA is the difference in corticosteroid absorbance in the absence and presence of β-CD, and X is the molar ratio API/β-CD. The stoichiometry of the inclusion complex was determined by the point at which the maximum deviation of the physical property was located on the graph.

## 3. Results and Discussion

In this study, micronization with the SAS technique was carried out to obtain corticosteroid inclusion complexes, which have shown benefits in many fields of application. In particular, the active ingredients selected, i.e., DEX and PRED, were micronized in the presence of a hydrophilic carrier to increase the relative rate of dissolution in water and, consequently, the bioavailability of the same within the human body. To that end, SAS micronization was carried out by varying different operating conditions to identify the most suitable ones. During the tests, the effect of pressure (P), the active substance/carrier ratio, and the total concentration (Ctot) of solutes (active substance and β-cyclodextrin) in the solvent used (DMSO) were evaluated. The experiments performed are summarized in Table 1, in which the morphology obtained, the average diameter of the particles (MD), and the standard deviation (SD) of the particle size distributions on a volumetric basis are indicated. In addition, the operating conditions investigated are shown, namely, the pressure, the total concentration of solutes in the injected solution (Ctot), and the active compound/carrier ratio.

### 3.1. Preliminary Test for DEX Micronization

A preliminary micronization test of pure DEX was carried out to assess the behavior of the active substance alone after SAS micronization. The following operating parameters were set: temperature of 40 °C, pressure of 90 bar, and injection of a solution with a concentration of DEX in DMSO equal to 10 mg/mL.

Upon opening the precipitation chamber, a small amount of powder was found on the filter; this meant that most of the injected drug had been extracted from the CO_2_/DMSO mixture. Therefore, this active substance was undoubtedly a candidate unsuitable to be micronized with the SAS technique.

**Figure 2:**
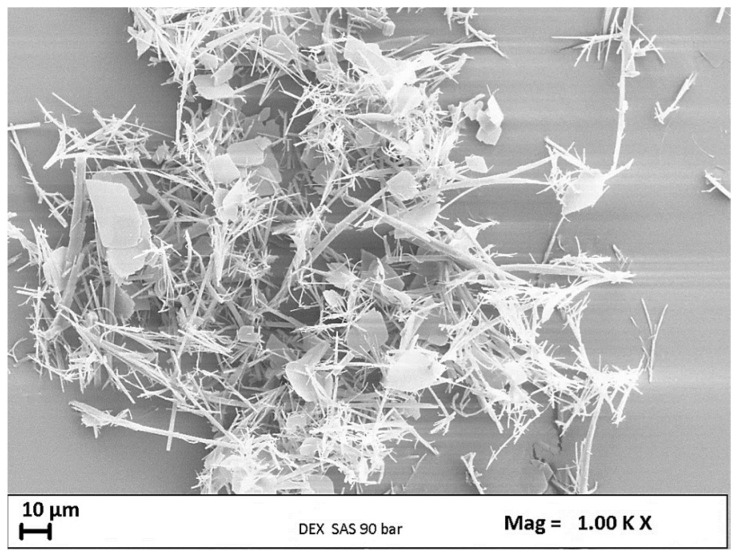
DEX crystals obtained from the SAS process at 40 °C and 90 bar.

The derisory amount of precipitated drug was analyzed through scanning electron microscopy. As can be seen from the image below (Figure 2), the powder precipitation occurred in the form of crystals.

### 3.2. DEX/β-Cyclodextrin

The behavior of the DEX/β-cyclodextrin system was studied by evaluating how operating parameters affect the morphology, size, and particle size distribution of the obtained particles. In particular, the effect of the active ingredient/carrier molar ratio was investigated, as was the effect of the total concentration of solutes in the solution injected into the precipitation chamber.

#### 3.2.1. Effect of the DEX/β-CD Ratio

To assess the effect of the API/carrier molar ratio, the total concentration in DMSO was set at 20 mg/mL and the pressure at 120 bar. The powder obtained at the end of the process was then compared using the API/carrier ratios of 1/1 mol/mol and 1/2 mol/mol.

**Figure 3:**
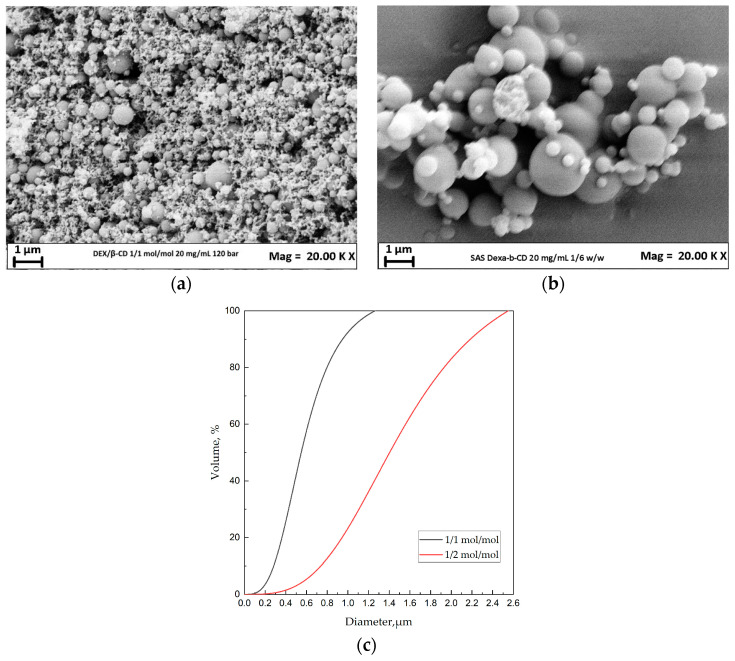
DEX/β-cyclodextrin precipitated at 40 °C, 120 bar, and 20 mg/mL. Effect of the molar ratio. (**a**) FESEM image of the particles obtained at 1/1 mol/mol; (**b**) FESEM image of the particles obtained at 1/2 mol/mol; (**c**) comparison of the cumulative volumetric PSDs.

In the FESEM images shown in Figure 3a,b, the production of microparticles can be observed in both cases. The average diameters for the two tests are 0.197 μm and 0.609 μm, respectively.

By comparing the cumulative volumetric distributions (Figure 3c) for various DEX/β-cyclodextrin molar ratios at a set pressure, temperature, and concentration, it was possible to observe that increasing the API/carrier molar ratio has a profound influence on the average size of DEX/β-cyclodextrin particles; in particular, the diameter tends to increase. This experimental evidence can be explained by considering the thermodynamics of the system. When a more significant amount of API is added to the mixture, the system’s critical point (MCP) moves to higher pressure values [29]. Therefore, in correspondence with the same pressure and temperature conditions, the expected morphology switched from sub-microparticles to microparticles.

#### 3.2.2. Effect of Concentration

The effect of the concentration was assessed by fixing the DEX/β-CD molar ratio at 1/2 mol/mol and the pressure equal to 120 bar. The concentration values tested were 20 mg/mL and 200 mg/mL.

The first test was conducted with a total concentration of 20 mg/mL. As already seen in Figure 3b, spherical microparticles were obtained.

**Figure 4:**
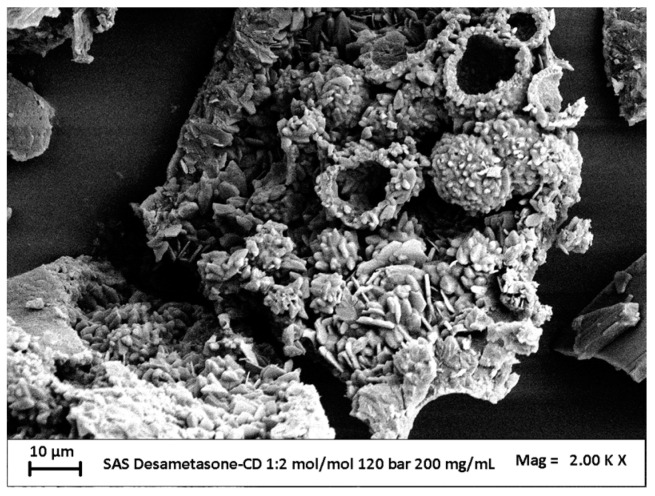
FESEM image of dexamethasone/β-cyclodextrin powders precipitated at 40 °C, 120 bar, 200 mg/mL, and 1/2 mol/mol.

In the test where we injected a solution with a total concentration of 200 mg/mL, only crystals and agglomerated expanded particles were obtained, as can be observed from the FESEM image reported in Figure 4. Therefore, it was impossible to evaluate the PSD and compare it with that obtained by precipitating the powders at 20 mg/mL.

### 3.3. Preliminary Test for PRED Micronization

**Figure 5:**
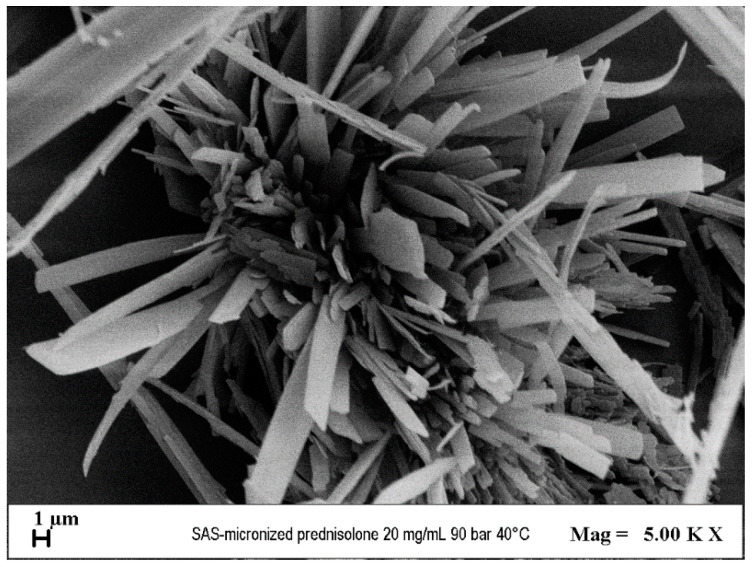
PRED crystals obtained from the SAS process at 40 °C and 90 bar.

Before proceeding with the SAS process tests, a preliminary test was carried out for the pure prednisolone with the following operational parameters: temperature of 40 °C and pressure of 90 bar, by injecting a solution into the precipitation chamber with an active substance concentration in DMSO of 20 mg/mL. At the end of the same test, it was observed that, both on the surface of the filter and on the inner walls of the chamber, there was no trace of precipitated or almost precipitated material. This made it possible to establish that prednisolone is soluble in the solvent–antisolvent mixture that forms in the precipitation chamber and is extracted during the process. Through electron microscope observation of the negligible amount of powder recovered at the end of the test, it was possible to detect the presence of large crystals (Figure 5).

### 3.4. Micronization of PRED/β-CD Complexes

PRED/β-CD system was studied by evaluating the effect of pressure, the drug/carrier molar ratio, and concentration on morphology and particle size.

#### 3.4.1. Effect of Pressure

**Figure 6:**
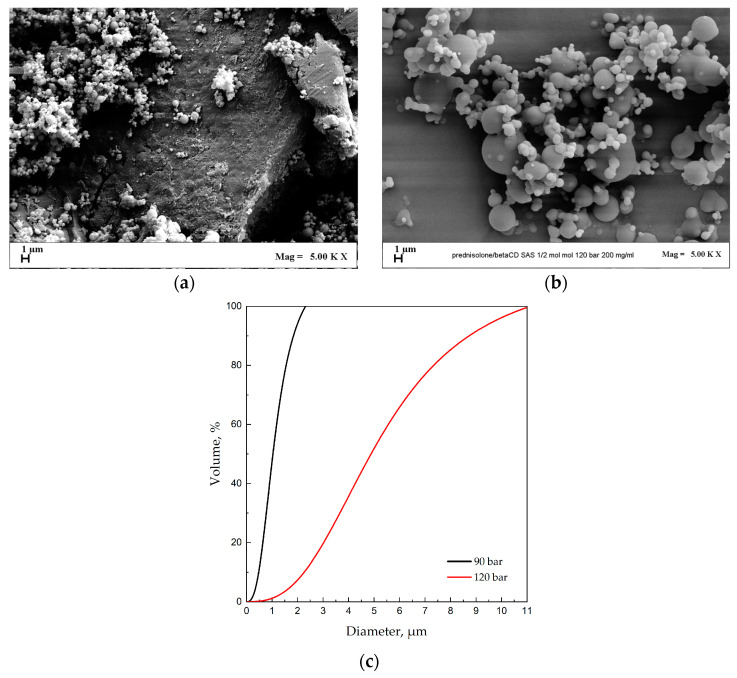
PRED/β-cyclodextrin precipitated at 40 °C, 1/2 mol/mol and 200 mg/mL. Effect of the pressure. (**a**) FESEM image of the particles obtained at 90 bar; (**b**) FESEM image of the particles obtained at 120 bar; (**c**) comparison of the cumulative volumetric PSDs.

The effect of pressure was evaluated by fixing the PRED/β-cyclodextrin ratio at 1/2 mol/mol and the total solute concentration at 200 mg/mL; however, the pressure varied from 90 bar to 120 bar. For the initial test, a pressure of 90 bar was used in the chamber. Analysis through FESEM microscopy of the resulting samples confirmed the production of both microparticles and large crystals, as visible in Figure 6a,b.

The average diameters were 0.435 μm and 2.414 μm, respectively, for the two tests.

As can be observed from Figure 6a,b, the increase in operating pressure leads to the formation of well-defined micrometric particles. Indeed, at 120 bar (Figure 6b), the complete disappearance of the crystalline form is observed; this can be explained by observing the thermodynamics of crystal formation at that specific pressure and temperature. Specifically, at 90 bar, the crystallization time is faster than the solvent removal time for this mixture, leading to the formation of large crystals and small particles precipitated onto them.

Overall, looking at Figure 6c, it can be observed that the increase in pressure leads to particles with a larger diameter but, simultaneously, the disappearance of the crystalline form.

#### 3.4.2. Effect of Molar Ratio PRED:β-CD

Another investigated parameter was the active principle/carrier molar ratio; to test this, two different values were used: 1/1 mol/mol and 1/2 mol/mol. The parameters set for each test were a pressure equal to 120 bar, a temperature of 40 °C, and a concentration of 200 mg/mL.

**Figure 7:**
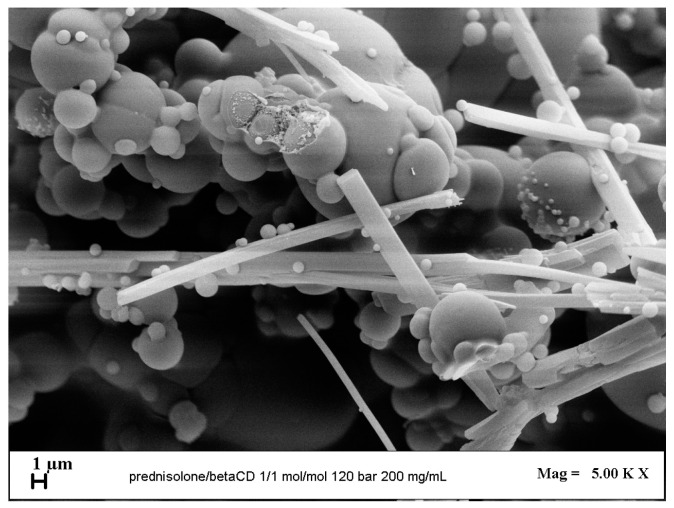
FESEM image of PRED/β-cyclodextrin powders precipitated at 40 °C, 120 bar, 200 mg/mL, and 1/1 mol/mol.

The first test was conducted with an active substance/carrier molar ratio of 1/1 mol/mol. In Figure 7, a high degree of coalescing microparticles and the presence of crystals can be observed. Therefore, the particle size distribution was not evaluated.

The second test, carried out with a 1/2 mol/mol PRED/β-cyclodextrin molar ratio, as shown in the previous section, led to the attainment of only microparticles, some of which were coalescing (Figure 6b).

Therefore, after the tests were carried out, it was possible to observe that the variation in the active principle/carrier molar ratio led to a significant alteration of the obtained morphology. When passing from the test at 1/1 mol/mol to the test at 1/2 mol/mol, the crystals disappear at the end of the process. Therefore, it can be concluded that the greater amount of active substance present in the test at 1/1 involved a shift of the MCP (mixture critical point) toward higher pressure values, and when working under the same operating conditions, the operating point fell within the miscibility gap.

#### 3.4.3. Effect of Total Concentration

The parameter investigated next was the effect of the total concentration; in particular, two different values were examined: 20 mg/mL and 200 mg/mL.

Meanwhile, the parameters left unchanged for testing were the molar ratio of 1/1 mol/mol (1/3 *w*/*w*), the pressure of 120 bar, and the temperature of 40 °C.

**Figure 8:**
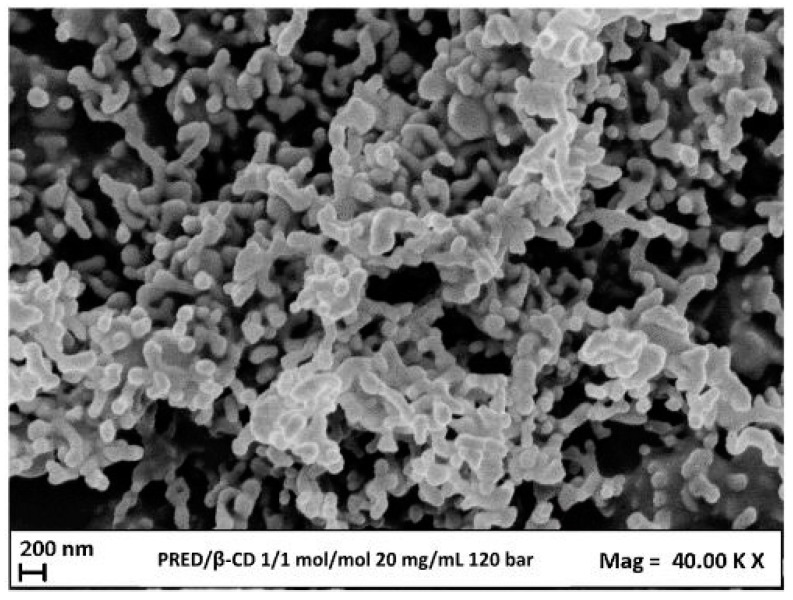
FESEM image of PRED/β-cyclodextrin SAS micronized powders produced at 120 bar, 20 mg/mL, and 1/2 mol/mol and at a temperature of 40 °C.

The first test was conducted with a 20 mg/mL concentration value. The FESEM image in Figure 8 showed the formation of coalescing microparticles. Due to coalescence, plotting the distribution of particle sizes was impossible.

The second test was then conducted at 200 mg/mL. In this case, as mentioned in the previous section, it is noted that no well-distinguished spherical particles were obtained. Figure 7 shows an evident irregularity in the shape and a high degree of coalescence beyond the presence of crystals.

Therefore, the variation in the concentration of solutes inside the solvent led to a remarkable alteration in the obtained morphology that can undoubtedly be explained by observing the thermodynamics of the considered system.

### 3.5. Determination of Encapsulation Yield

The primary indicator of a successful complexation is the encapsulation yield of the active ingredient in SAS powder. Table 2 provides an overview of the results obtained from various SAS samples.

### 3.6. Characterization

Fourier transform spectroscopic analyses (FT-IRs) were carried out to obtain information on the formation of inclusion complexes and to verify if there were interactions between the functional group of active ingredients and the carrier.

**Figure 9:**
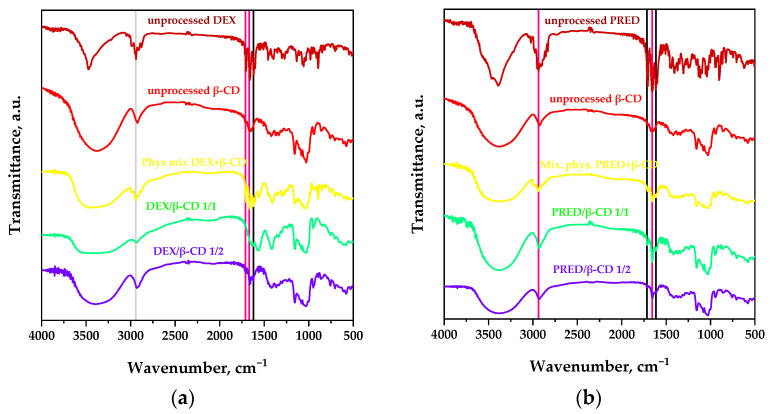
FT-IR spectra comparison among raw powder samples, API/β-cyclodextrin physical mixtures, and API/β-cyclodextrin SAS processed samples for (**a**) dexamethasone and (**b**) prednisolone. Pink lines represent the typical peaks of the API found in both physical mixtures and SAA powder spectra. In contrast, black lines indicate API-specific peaks observed with reduced intensity in the SAA powder spectra.

Figure 9a,b show and compare the FT-IR spectra of unprocessed active ingredients, the pure carrier, physical mixtures, and powders obtained using the SAS process. In particular, the FT-IR spectrum of β-CD displays a broad absorption band between 3571 cm^−1^ and 3220 cm^−1^, corresponding to the -OH group stretching; bands at about 2928 cm^−1^ and 1158 cm^−1^, which correspond to the stretching of the bonds -CH_2_ and -C-C; and a peak at 1029 cm^−1^, attributable to the bending of the -O-H group [30].

The spectrum of DEX alone (Figure 9a) shows two bands at about 3522 cm^−1^ and 3381 cm^−1^ that correspond to the stretching of -OH and -OH…H (hydrogen bond), respectively; a peak at 2935 cm^−1^ corresponding to the stretching of the bond -C-H; three additional peaks at about 1702 cm^−1^, 1658 cm^−1^, and 1620 cm^−1^ for stretching the bond -C=O; and a broad band at 1261 cm^−1^ due to stretching of the -C-F bond [31,32].

With regards to the spectrum of pure PRED (Figure 9b), it is possible to observe a band in the range of 3200–3500 cm^−1^ characteristic of the -OH group, a peak at about 2947 cm^−1^ due to the -C-H group, and three very intense peaks at 1708 cm^−1^, 1654 cm^−1^, and 1614 cm^−1^ due to the -C=O carboxylic groups [31].

Looking at Figure 9a, it is clear that the characteristic bands of the carrier predominate in the spectra of processed powders and the physical mixture. In addition, the vertical line in black highlights the characteristic peak of DEX in the two processed SAS samples and the physical mixture (at 1620 cm^−1^), while the vertical lines in pink show the characteristic peaks of the drug (at 1702 cm^−1^ and 1658 cm^−1^), which are present on the test spectrum 1/2 mol/mol and not on the test spectrum 1/1 mol/mol. This indicates that using an active ingredient/carrier molar ratio equal to 1/1 increases the complexation degree.

From Figure 9b, where PRED spectra are shown, it can be observed that the physical mixture and processed powders have a more significant predominance of the characteristic bands of the carrier, demonstrating that the guest molecule (prednisolone) is embedded in the host molecule cavity (β-CD). Hence, the carrier hides its characteristic bands.

In addition, the characteristic peaks of the drug detectable in SAS powders and the physical mixture (at 2947 cm^−1^ and 1654 cm^−1^) are indicated by vertical lines in pink, while through vertical lines in black, the characteristic peaks of the drug at 1708 cm^−1^ and 1614 cm^−1^ are indicated, which are absent from SAS powder with an active principle/carrier ratio of 1/2 mol/mol (this is since from the test at 1/1 mol/mol, as shown in the FESEM images in Figure 7, both crystals and microparticles were obtained).

In the spectra, a lower intensity of the peaks characteristic of the drug can also be noted, indicating that prednisolone formed partial inclusion complexes with β-CD (that is, only a tiny part of prednisolone was outside of the cavity).

**Figure 10:**
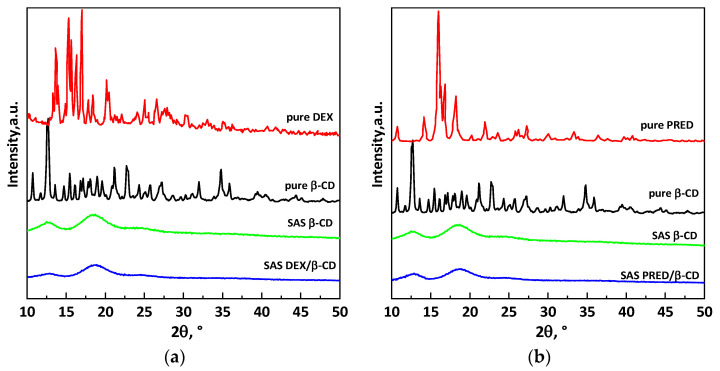
XRD patterns comparison among raw powder samples, β-cyclodextrin SAS processed sample, and API/β-cyclodextrin SAS processed samples for (**a**) dexamethasone and (**b**) prednisolone.

The crystalline state of the powders was evaluated through an XRD analysis. Figure 10 shows the plots for unprocessed corticosteroids, unprocessed and SAS-processed β-CD, and API/β-CD complexes for dexamethasone (Figure 10a) and prednisolone (Figure 10b). For both active compounds, it is possible to note that the diffractograms of unprocessed DEX, PRED, and β-CD show the strong, sharp peaks characteristic of each material, demonstrating their crystalline nature. On the contrary, materials processed using the SAS technique have an amorphous structure. This change in the solid phase has been attributed in the literature to an interaction between the drug and β-CD due to the formation of inclusion complexes [33]. In detail, the characteristic peaks of the drugs disappeared in the XRD patterns of the SAS complexes since the molecules of the active ingredients are incorporated and hidden in the cavity of the amorphous CD. Therefore, the XRD patterns suggest that inclusion complexes formed, given the absence of strong peaks that generally characterize co-crystalline materials.

The pattern of β-CD precipitated via SAS without any corticosteroid has been inserted in Figure 10 to strengthen the hypothesis of the formation of inclusion complexes. The pattern of the coprecipitate is very similar, but not identical, to that of the β-CD both in the case of DEX (Figure 10a) and in the case of PRED (Figure 10b); indeed, the ratio between the intensities of the two residual peaks differs between the pattern relating to the API/β-CD powder and the β-CD pattern. This could be due to new interactions (weak bonds) created between the corticosteroid and the cyclodextrin and, therefore, can be considered further evidence of the formation of an inclusion complex.

Once the presence of the active substance inside the samples produced using the SAS technique was verified, using UV–vis spectroscopy, the dissolution profiles of the pure active principle and the microparticles obtained with the SAS technique were compared, using PBS (a saline phosphate buffer) at pH 7.4 and a temperature of 37 °C as the dissolving medium. The objective was also to assess the effect of carrier presence on the dissolution of the active substance.

**Figure 11:**
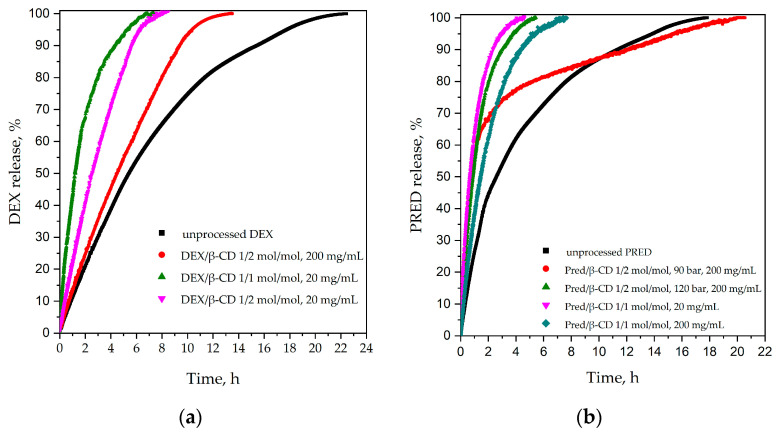
Comparison of the release kinetics acquired from pure API and API/β-cyclodextrin samples, obtained via SAS process, for (**a**) DEX and (**b**) PRED.

Figure 11 compares the release profiles obtained for both active principles.

Figure 11a shows the release kinetics of pure DEX and those obtained from particles produced with the SAS technique. From Figure 11a, it can be observed that SAS microparticles can make the drug release about three times faster than pure DEX. The unprocessed DEX has release times in an aqueous environment of about 22 h, while all samples release in much better times of about 8 h. That is, apart from the sample obtained using a molar ratio of 1/2 mol/mol and a total concentration of 200 mg/mL, which had a release time of approximately 14 h. This could be explained by observing the sample’s morphology, which was characterized by large drug crystals and agglomerated expanded particles. Then, from our comparison of kinetics, shown in Figure 11a, it is possible to observe that the release time tended to decrease with the decrease in the molar ratio of the active substance/vector. In particular, the sample with the lowest release time was the one with a molar ratio of 1/1 and a concentration of 20 mg/mL.

Finally, the dissolution time of pure PRED was about 18 h. In this case, it can be observed that sample processing with the SAS technique can effectively accelerate the release of the active substance for up to 4 h. But even this sample obtained using a molar ratio of 1/2, a total concentration of 200 mg/mL, and a pressure of 90 bar (red line) had longer release times compared to pure PRED. This phenomenon can be explained by looking at the sample’s morphology, shown in Figure 6a, where the formation of large crystals of drug covered by the cyclodextrin microparticles can be observed. In addition, the kinetic comparison in Figure 11b shows that the release time decreased significantly for samples produced with a molar ratio of 1/1 active substance/vector. In particular, the most efficient release profile was obtained in the case of the sample with a 1:1 molar ratio and a total concentration of 20 mg/mL (pink line); the drug was released in about 2 h.

Therefore, the best release profile for both active ingredients was obtained from the sample produced using an active ingredient/carrier molar ratio of 1/1, total concentration of 20 mg/mL, pressure of 120 bar, and temperature of 40 °C. These results could indicate the effective complexation of the active substance in the β-cyclodextrin cavity using a 1/1 molar ratio.

The in vitro release profiles corresponding to the optimized conditions were fitted with different kinetic models: Korsmeyer–Peppas, Peppas–Sahlin, Higuchi, and Weibull. The equations of the models, the values of the constants, and the coefficients of determination (R^2^) are shown in Table 3.

For both the DEX and PRED release curves, the highest R^2^ values were observed in the Weibull model. Indeed, the Weibull model is an empirical function widely applied to the analysis of dissolution and release studies involving nanoparticulate drug systems [34,35]. In this model, the dissolution curve is described in terms of scale (a) and curve shape parameter (b). The latter parameter characterizes the shape of the curve as exponential (b = 1), as sigmoid S-shaped with upward curvature (b > 1), or as parabolic with a high initial slope and a consistent exponential character (b < 1) [36]. The exponent b also has a physical meaning, being an indicator of the mechanism of transport of the drug through the nanoparticulate matrix [37]. A value of b ≤ 0.75 indicates Fickian diffusion, while 0.75 < b < 1 indicates a combined release mechanism involving Fickian diffusion and swelling-controlled transport [38]. The values of b reported in Table 3 are equal to 0.916 and 0.931 for DEX and PRED, respectively, indicating a combined release mechanism.

### 3.7. Determination of Stoichiometries of Inclusion Complexes

**Figure 12:**
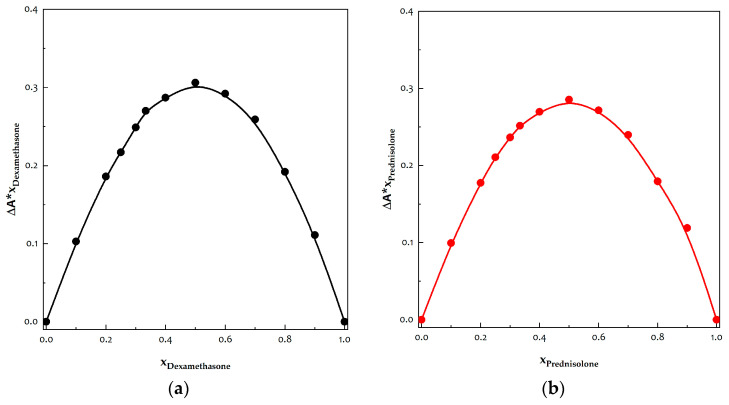
Diagram illustrating Job plot for determining stoichiometry. The inclusion complex’s stoichiometry is indicated by the maximum position: (**a**) dexamethasone and (**b**) prednisolone.

The stoichiometric ratio of guest to host molecules was determined based on the X value corresponding to the maximum deviation. As suggested by Saha et al. [39], the inclusion complex’s stoichiometry can be assigned accordingly: a ratio of 1/2 for guest molecule to host molecule is indicated if the maximum occurs at X = 0.33; the equimolar ratio at X = 0.5; a ratio of 2/1 at X = 0.66; and so forth. In the case of DEX and PRED, the maximum for each plot was found at X = 0.5, as reported in Figure 12, implying a 1/1 stoichiometry between the API and β-cyclodextrin in both cases.

The 1/1 stoichiometric ratio represents the optimal formation ratio for a stable inclusion complex in these systems, regardless of the method by which the complexes are formed. This ratio, determined by Job’s method, depends solely on the cyclodextrin type and the active principles considered.

## 4. Conclusions

The SAS process has been shown to be exceptionally efficient in producing inclusion complexes using DEX and PRED as active ingredients in the form of microparticles, thus using a water-soluble matrix that increases the bioavailability of the two corticosteroids. The powders obtained do not always have a well-defined spherical morphology, but under the optimal operating conditions that are found to be 1/1 molar ratio, pressure of 120 bar, and total concentration of 20 mg/mL, for DEX, spherical microparticles are obtained with an average diameter of 0.197μm. Meanwhile, for PRED, the optimal conditions under which only well-defined microparticles are obtained are found to be a molar ratio equal to 1/2, concentration of 20 mg/mL, and pressure of 120 bar; under these conditions, the average diameter of the particles obtained is 2.414 μm.

The formation of inclusion complexes was demonstrated through FT-IR and XRD analyses: FT-IR spectra showed that some peaks decreased or shifted, whereas XRD patterns confirmed the changes in the solid state. To test the effectiveness of the powders obtained through the SAS process, in vitro release tests were tested to simulate the physiological environment; these tests showed that the active ingredients contained in the powders processed using the SAS technique had much shorter release times. The best release profile for both APIs was obtained by setting the molar ratio to 1/1 and the total concentration to 20 mg/mL. The Weibull model fitted the release curves with R^2^ parameters of 0.9993 and 0.9999 for DEX and PRED, respectively; the Weibull shape parameter, for both the corticosteroid/β-CD inclusion complexes, was compatible with a combined release mechanism involving Fickian diffusion and controlled release.

To verify the formation of the inclusion complex, the Job method was used to identify its stoichiometry. The method indicated that the stoichiometric complexation ratio was 1:1 for both active substances; this value represents stoichiometric complexation of a stable complex that can occur both before and during the process.

In conclusion, the SAS process is promising for the effective formation of inclusion complexes that increase the bioavailability of the two active ingredients (DEX and PRED) and allow an accelerated release of drugs. Therefore, the results obtained are important for guiding future efforts to develop innovative pharmaceutical formulations.

## Figures and Tables

**Table 1:** SAS tests carried out to produce β-CD-based inclusion complexes (MP = microparticles, cMP = coalescing microparticles, C = crystals, AGG = agglomerates).

API	API/β-CD (mol/mol)	P (bar)	C_tot_ (mg/mL)	Morphology	MD ± SD (μm)
/	0/1	120	100	MP	1.731 ± 0.48
DEX	1/0	120	10	C	-
	1/1	120	20	MP	0.197 ± 0.18
	1/2	120	20	MP + cMP	0.609 ± 0.42
		120	200	C + AGG	-
PRED	1/0	90	20	C	-
	1/1	120	20	cMP	0.131 ± 0.07
		120	200	cMP + C	4.19 ± 4.03
	1/2	90	200	cMP + C	0.435 ± 0.30
		120	200	cMP + MP	2.14 ± 1.43

**Table 2:** Encapsulation yield for some samples.

API	API:β-CD (mol:mol)	P (bar)	C_tot_ (mg/mL)	Encapsulation Yield (%)
DEX	1/1	120	200	93
	1/2	120	20	87
			200	57
PRED	1/1	120	20	89
			200	78
	1/2	90	200	55
		120	200	68

**Table 3:** Release kinetic modeling for the particles obtained at an active ingredient/carrier molar ratio of 1/1, total concentration of 20 mg/mL, pressure of 120 bar, and temperature of 40 °C.

Model and Equation	a	b	c	R^2^
Dexamethasone				
Korsmeyer–Peppas y = a × t^b^	44.811 ± 0.279	0.452 ± 0.279		0.9667
Peppas–Sahlin y = a × t^b^ + c × t^2b^	50.331 ± 0.122	0.729 ± 0.003	−6.430 ± 0.034	0.9972
Weibull y = 100 × (1 − exp(−a × t^b^))	1.898 ± 0.007	0.916 ± 0.004		0.9993
Higuchi y = a × t^0.5^	42.041 ± 0.138			0.9602
Prednisolone				
Korsmeyer–Peppas y = a × t^b^	60.397 ± 0.297	0.403 ± 0.005		0.9540
Peppas–Sahlin y = a × t^b^ + c × t^2b^	60.397 ± 0.298	0.403 ± 0.005	4.115 × 10^−16^ ± 0	0.9546
Weibull y = 100 × (1 − exp(−a × t^b^))	1.070 ± 0.002	0.931 ± 0.002		0.9999
Higuchi y = a × t^0.5^	56.032 ± 0.263			0.9190

## Data Availability

Data are contained within the article.
